# Novel aminoacylases from *Streptomyces griseus* DSM 40236 and their recombinant production in *Streptomyces lividans*

**DOI:** 10.1002/2211-5463.13723

**Published:** 2023-11-01

**Authors:** Gerrit Haeger, Johanna Probst, Karl‐Erich Jaeger, Johannes Bongaerts, Petra Siegert

**Affiliations:** ^1^ Institute of Nano‐ and Biotechnologies Aachen University of Applied Sciences Jülich Germany; ^2^ Institute of Molecular Enzyme Technology Heinrich Heine University Düsseldorf Jülich Germany; ^3^ Institute of Bio‐ and Geosciences IBG‐1: Biotechnology Forschungszentrum Jülich GmbH Jülich Germany

**Keywords:** acyl amino acids, recombinant expression, *Streptomyces griseus*, *Streptomyces lividans*, α‐aminoacylase, ε‐lysine acylase

## Abstract

Amino acid‐based surfactants are valuable compounds for cosmetic formulations. The chemical synthesis of acyl amino acids is conventionally performed by the Schotten–Baumann reaction using fatty acyl chlorides, but aminoacylases have also been investigated for use in biocatalytic synthesis with free fatty acids. Aminoacylases and their properties are diverse; they belong to different peptidase families and show differences in substrate specificity and biocatalytic potential. Bacterial aminoacylases capable of synthesis have been isolated from *Burkholderia*, *Mycolicibacterium*, and *Streptomyces*. Although several proteases and peptidases from *S. griseus* have been described, no aminoacylases from this species have been identified yet. In this study, we investigated two novel enzymes produced by *S. griseus* DSM 40236^T^. We identified and cloned the respective genes and recombinantly expressed an α‐aminoacylase (EC3.5.1.14), designated SgAA, and an ε‐lysine acylase (EC3.5.1.17), designated SgELA, in *S. lividans* TK23. The purified aminoacylase SgAA was biochemically characterized, focusing on its hydrolytic activity to determine temperature‐ and pH optima and stabilities. The aminoacylase could hydrolyze various acetyl amino acids at the N_α_‐position with a broad specificity regarding the sidechain. Substrates with longer acyl chains, like lauroyl amino acids, were hydrolyzed to a lesser extent. Purified aminoacylase SgELA specific for the hydrolysis of N_ε_‐acetyl‐l‐lysine was unstable and lost its enzymatic activity upon storage for a longer period but could initially be characterized. The pH optimum of SgELA was pH 8.0. While synthesis of acyl amino acids was not observed with SgELA, SgAA catalyzed the synthesis of lauroyl‐methionine.

## Graphical abstract

Two novel aminoacylases were identified in Streptomyces griseus DSM 40236^T^. The genes for the putative aminoacylases, designated SgAA and SgELA, have been cloned for expression in *Streptomyces lividans* TK23. The resulting strains have been cultivated in bioreactors for recombinant expression, and the two aminoacylases have been purified and biochemically characterized. With SgAA, synthesis of lauroyl‐methionine was achieved.

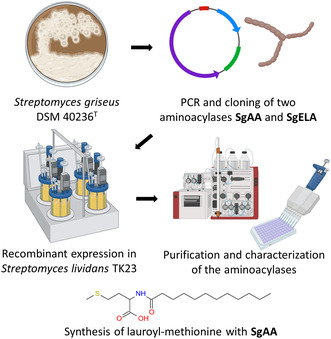

**Abbreviations**
EDTA: ethylenediaminetetraacetic acid
ELSD: evaporative light scattering detector
HiDapE: N‐succinyl‐l,l‐diaminopimelic acid desuccinylase from *Haemophilus influenzae*
LB: Lysogeny Broth
MS: mass spectrometry
MWCO: molecular weight cutoff
PAGE: polyacrylamide gel electrophoresis
PCR: polymerase chain reaction
SamAA: aminoacylase from *S. ambofaciens*
SamELA: ε‐lysine acylase from *S. ambofaciens*
SDS: sodium dodecyl sulfate
SgAA: aminoacylase from *S. griseus*
SgELA: ε‐lysine acylase from *S. griseus*
SmAA: aminoacylase from *S. mobaraensis*
SmELA: ε‐lysine acylase from *S. mobaraensis*
TB: Terrific Broth
Tris: tris(hydroxymethyl)aminomethane
UV: ultraviolet

N‐acyl‐l‐amino acids are valuable compounds and are used as biosurfactants in cosmetic products. Several advantages of N‐acyl‐l‐amino acids compared with conventional surfactants like laureth sulfates include more skin‐friendly properties, a low inflammatory potential and biodegradability [1]. Long‐chain acyl amino acids can even have physiological function due to their structural resemblance to endocannabinoids [2], and acetyl amino acids can be found in the brain [3, 4]. With the motivation to establish a biocatalytic production of these biosurfactants, aminoacylases have been investigated and show high potential to replace the environmentally harmful Schotten–Baumann synthesis. Aminoacylases that were shown to be capable of synthesis of N‐acyl‐l‐amino acids have been identified in *Streptomyces* [5, 6, 7], *Mycolicibacterium* [8], *Burkholderia* [9], and pig liver [10, 11].

Several α‐aminoacylases with broad substrate spectrum have been identified that belong to the family of M20A peptidases. Members of this family share a similar fold and three‐dimensional structure, albeit with low homology of amino acid sequences. Often, these enzymes are dimeric [12]. The protein structure can be divided into a catalytic and a dimerization domain, or lid domain for monomeric M20A peptidases. The binuclear active site is composed of metal‐binding and catalytic residues. The metal ions are bound by conserved residues, namely two histidines, two glutamic acids, and aspartic acid. The catalytic residues are aspartic acid and glutamic acid, and the latter acts as a general base catalyst in hydrolysis or synthesis. Furthermore, a conserved histidine contributes to the formation of an oxyanion hole, stabilizing the tetrahedral reaction intermediate [13]. This histidine is located at the tip of the dimerization domain and reaches into the active site of the opposing dimer. In monomeric members of the M20A family, the lid domain structurally represents a doubled dimerization domain, and the histidine protrudes into the active site of the same monomer [14, 15]. The aminoacylases SmAA and SamAA, both nonpeptidase homologs of the M20A family, have been isolated from *S. mobaraensis* [16] and *S. ambofaciens* [5, 17], respectively. Especially SamAA showed a high potential for acylation of various amino acids. For SmAA, no synthesis has been shown yet [16]. The aminoacylase from *M. smegmatis* (MsAA) is homologous to SmAA and SamAA and has been shown to catalyze acylation of amino acids as well [8]. Further aminoacylase members of the M20A peptidase family are pAcy1 and hAcy1 from porcine and human liver, respectively [18, 19]. The homologous N‐succinyl‐l,l‐diaminopimelic acid desuccinylase from *Haemophilus influenzae* (HiDapE) has also been extensively investigated providing insights into the characteristics of this family [13, 20, 21, 22, 23].

From *S. mobaraensis* IFO 13819, *S. coelicolor* A3 (2) and *S. ambofaciens* ATCC 23877, ε‐lysine acylases have been identified as well, designated SmELA [6], ScELA [24], and SamELA [5], respectively. While SmAA and SmELA have been recombinantly expressed in *S. lividans* TK24 [16, 25], SamAA and SamELA were obtained from the natural producer, despite efforts to express the enzyme in *E. coli* Origami B(DE3) [26]. The recombinant expression of ScELA with *E. coli* JM109 and *C. glutamicum* YDK010 has been described in a patent [24]. The synthetic potential of SmELA is extraordinarily high, with N_ε_‐lauroyl‐l‐lysine being synthesized with conversion rates reaching 100% [25]. Using ScELA‐containing cell extract from recombinant *C. glutamicum* to synthesize N_ε_‐lauroyl‐l‐lysine, conversions reaching 100% were also achieved [24]. In contrast to the aminoacylases belonging to the M20A family, ε‐lysine acylases have not been investigated as extensively, and no structure has been published. Further ε‐lysine acylases or ε‐peptidases have been identified from *Achromobacter pestifer* [27, 28] and avian kidney [29] but the genes have not been cloned or sequenced. Classification of SmELA to the YtcJ‐like metal‐dependent amidohydrolase family has been described [25].

Several hydrolytic enzymes have been described from *S. griseus* that are highly interesting for technical applications [30, 31, 32, 33]. A commercial preparation of proteinases and peptidases from this species is available under the name Pronase® [34]. An aminopeptidase from *S. griseus* was described, which belongs to the M28 family of metallopeptidases [35]. However, extracellular peptidases from *S. griseus* could not hydrolyze chloroacetyl amino acids [36]. Surprisingly, no aminoacylases have yet been described from *S. griseus*. The goal of this study was to identify, recombinantly express, and characterize homologous aminoacylases from *S. griseus* as a reference to the enzymes identified in *S. mobaraensis* and *S. ambofaciens* and to extend the scope of characterized aminoacylases. We searched for homologs in *S. griseus* DSM 40236^T^ (ATCC 23345) as the type strain of this species and identified two enzymes, designated SgAA and SgELA. The respective genes were cloned for recombinant expression in *S. lividans* TK23, and the enzymes were purified and biochemically characterized. Finally, we investigated the acylation activity of the enzymes using all proteinogenic amino acids as substrates.

## Materials and methods

### Chemicals and reagents

Cultivation media, metal salts, amino acids, Tris (tris(hydroxymethyl)aminomethane), and solvents were from Carl Roth (Karlsruhe, Germany). Acetyl amino acids were purchased from Sigma‐Aldrich (Taufkirchen, Germany), and other acyl amino acids were chemically synthesized as described previously [8]. Molecular biology reagents were from Thermo Fisher Scientific (Langerwehe, Germany). Oligonucleotide synthesis and DNA sequencing were ordered from Eurofins Genomics (Ebersberg, Germany). Strep‐Tactin columns were purchased from IBA (Göttingen, Germany). Reagents for native PAGE were obtained from SERVA Electrophoresis (Heidelberg, Germany). The EZ Nin reagent was from Biochrom (UK). Remaining chemicals were from Sigma‐Aldrich.

### Strains and cultivation media

For cloning and plasmid maintenance, *E. coli* DH5α (Thermo Fisher Scientific, USA) was used and grown in LB medium. *S. griseus* DSM 40236^T^ was grown in liquid LB medium for isolation of genomic DNA. Heterologous expression was performed with *S. lividans* TK23 (spc‐1 SLP2^−^ SLP3^−^) [37] and *E. coli* BL21(DE3; Thermo Fisher Scientific, USA), *E. coli* BL21(DE3) pGro7 (transformed with GroEL/S overexpression plasmid from Takara Bio Europe, France), and *E. coli* ArcticExpress (DE3; Agilent Technologies, Santa Clara, CA, USA). Tryptic Soy Broth (TSB, MP Biomedicals, Eschwege, Germany) agar plates were used for strain maintenance and cultivation of *S. lividans* TK23. Shake flask cultures of *S. lividans* TK23 for expression and protoplast transformation were grown YEME medium [0.3% yeast extract, 0.5% peptone, 0.3% malt extract, 1% glucose, 34% sucrose, 0.2% magnesium chloride hexahydrate, all (w/v), and 25 mL of a 20% glycerol solution]. For bioreactor cultivations of *S. lividans* TK23, a fermentation medium (0.5% yeast extract, 3% TSB, 0.75% glucose, 0.3% malt extract, 10% sucrose 0.1% magnesium chloride hexahydrate) was developed. Oatmeal agar plates (20 g·L^−1^ ground oatmeal, 10 g·L^−1^ malt extract, 5 g·L^−1^ yeast extract, 20 g·L^−1^ agar) were used as sporulation plates. Preparation of spore suspensions for liquid media inoculation was performed by adding 10 mL of 0.1% Tween 80 (Carl Roth)/0.9% NaCl solution to the oatmeal agar plate. The suspension was filtered through glass wool (Carl Roth) to separate mycelium from the spores. The spore suspension was centrifuged at 4000 ***g*** for 10 min, and the pellet was resuspended in 0.9% NaCl solution. Expression cultures of *E. coli* BL21(DE3) were grown in Terrific Broth medium (TB; 2% tryptone from casein, 2.4% yeast extract, 25 mm NaH_2_PO_4_, 25 mm KH_2_PO_4_, 50 mm NH_4_Cl, 2 mm MgSO_4_, 5 mm Na_2_SO_4_, 0.5% glycerol (v/v), and 0.05% glucose).

### Database searches and sequence analysis

Homologs of aminoacylases SmAA, SamAA, SmELA, and SamELA were searched in *S. griseus* DSM 40236^T^ using the BLASTp service from NCBI (https://blast.ncbi.nlm.nih.gov/) [38]. Pairwise protein sequence alignment was conducted with the Needleman–Wunsch algorithm using the EMBOSS Needle tool from EMBL‐EBI (https://www.ebi.ac.uk/Tools/psa/emboss_needle/) [39]. Multiple protein sequence alignment was performed with the T‐Coffee algorithm (https://www.ebi.ac.uk/Tools/msa/tcoffee/) [40], and the results were visualized using ESPript 3.0 (https://espript.ibcp.fr/ESPript/ESPript/) [41]. The classification of protein sequences in the MEROPS system was performed by MEROPS BLAST [42].

### Cloning of aminoacylase genes from *S. griseus* DSM 40236^T^

For isolation of genomic DNA, *S. griseus* DSM 40236^T^ was grown and DNA was isolated with the innuPREP Bacteria DNA Kit (Analytik Jena, Jena, Germany) according to the manufacturer's instructions. Polymerase chain reaction (PCR) was performed on genomic DNA to amplify aminoacylase gene sequences using Phusion High‐Fidelity PCR Master Mix with GC‐buffer. The genes for SgAA and SgELA were amplified using primer pairs P1 (ATGAGCGAGAGCAGCACGGG) & P2 (TCAGGAGTGGTCGATGAACCGG) and P3 (ATGAGCCAGAGCACCGCCC) & P4 (TCACTCGTTCGGTCGCACGTAG), respectively. Further PCRs were performed to attach sequences for a Strep‐tag II (WSHPQFEK) with a linker (SG), either N‐ or C‐terminally, and restriction sites. For SgAA NTag, primers P5 (CGCAGTTCGAGAAGTCCGGCATGAGCGAGAGCAGCACGGG) & P6 (ATCGAATTCTCAGGAGTGGTCGATGAACCGG) and P7 (GATGCTAGCATGTGGTCCCACCCGCAGTTCGAGAAGTCCGGC) & P6 were used. For SgAA CTag, P8 (GATGCTAGCATGAGCGAGAGCAGCACGGG) & P9 (TGCGGGTGGGACCAGCCGGAGGAGTGGTCGATGAACCGGTCG) and P8 & P10 (ATCGAATTCtcaCTTCTCGAACTGCGGGTGGGACCAGCC) were used. For SgAA without affinity tag, P8 & P6 were used. For SgELA NTag, P11 (CGCAGTTCGAGAAGTCCGGCATGAGCCAGAGCACCGCCC) & P12 (ATCGAATTCTCACTCGTTCGGTCGCACGTAG) and P7 & P12 were used. For SgELA CTag, P13 (GATGCTAGCATGAGCCAGAGCACCGCCC) & P14 (TGCGGGTGGGACCAGCCGGACTCGTTCGGTCGCACGTAGACC) and P13 & P10 were used. For SgELA without affinity tag, P13 & P12 were used. The genes were cloned using NheI and EcoRI restriction sites and T4 ligase into pGH01. The pEM4‐based [43] plasmid pGH01 is a shuttle vector for *E. coli* and *S. lividans* and carries the ermE* promoter from *S. erythraea* for heterologous expression [44]. The plasmid conveys resistances against ampicillin and thiostrepton for *E. coli* and *S. lividans*, respectively. The resulting plasmids were designated pGH01 SgAA (NTag/CTag/noTag) and pGH01 SgELA (NTag/CTag/noTag).

The aminoacylases from *S. griseus* DSM 40236^T^ were also cloned for expression in *E. coli*. For this, the encoding nucleotide sequences were deduced and codon‐optimized according to the *E. coli* codon usage. Sequences encoding for the Strep‐tag II and linker were attached to both termini as well. The resulting DNA sequence was commercially ordered and synthesized by GeneArt (Thermo Fisher Scientific, USA). As described previously, the genes were amplified using primers with BsaI overhangs for Golden Gate cloning into pET28‐eforRED [8]. The primers used for amplification were P14 (GGTCTCCCATGTGGAGTCATCCTCAATTCGAAAAATCC) and P15 (GGTCTCTCTCAGCTATGATCAATAAAGCGATCCAGCACG) for SgAA NTag, P16 (GGTCTCCCATGAGCGAAAGCAGCACCGG) and P17 (GGTCTCTCTCATTTTTCGAATTGAGGATGACTCCATCC) for SgAA CTag, P16 and P15 for SgAA without tag, P14 and P18 (GGTCTCTCTCATTCATTCGGACGAACATAAACGGTCTG) for SgELA NTag, P19 (GGTCTCCCATGAGCCAGAGCACCGCAC) and P17 for SgELA CTag, P19 and P18 for SgELA without tag.

### Transformation of *S. lividans* TK23

Protoplast transformation of *S. lividans* TK23 was performed according to the standard procedure presented by Kieser *et al*. [37]. Thiostrepton (Merck Millipore, Darmstadt, Germany) was used at 30 μg·mL^−1^ for selection. Isolation of plasmid DNA to verify successful transformation from *S. lividans* TK23 was performed with modifications according to the protocol of Thompson *et al*. [45]. A spore suspension was inoculated into 25 mL of YEME medium, and the culture was incubated at 180 rpm and 30 °C for 3 days. The cells were centrifuged at 8000 ***g*** for 2 min and washed in 10% sucrose, 100 mm glucose and 25 mm Tris–HCl pH 7.0. After another centrifugation step, the pellet was incubated in the above buffer containing 1 mg·mL^−1^ lysozyme (SERVA Electrophoresis, Germany) and 100 μg·mL^−1^ RNAse (Carl Roth, Germany) for 1 h at 37 °C followed by plasmid purification with the GeneJET Plasmid‐Miniprep‐Kit (Thermo Fisher Scientific, USA).

### Recombinant expression in *E. coli*

Recombinant expression in *E. coli* was conducted with *E. coli* BL21(DE3), *E. coli* BL21(DE3) pGro7, and *E. coli* ArcticExpress(DE3) as previously described [8]. TB medium was used for growth. For induction with isopropyl β‐d‐thiogalactoside (IPTG), 1 mm IPTG was added at an OD_600_ of 0.5 and cells were harvested 4 h after induction (30 °C or 37 °C). For autoinduction, 0.2% (w/v) lactose was added to the TB medium, and the cultures were grown for 24 h at 20 °C or 30 °C. When *E. coli* BL21(DE3) pGro7 was used, 0.5 mg·mL^−1^ arabinose was added to the medium. *E. coli* ArcticExpress (DE3) was first cultured for 3 h or 6 h at 30 °C and subsequently grown at 12 °C for further 24 h. No antibiotics were used for this strain. The cells were harvested at 4000 ***g*** and 4 °C for 30 min.

### Recombinant expression in *S. lividans* TK 23

First, recombinant expression was performed in shake flasks. Recombinant *S. lividans* TK23 was cultivated in 500 mL baffled flasks containing 100 mL YEME medium complemented with 10 μg·mL^−1^ thiostrepton and 2 μL polyethylene glycol (PEG 2000). The medium was inoculated with a fresh spore suspension and a starting OD_600_ of 0.02. For expression, cultures were grown at 30 °C on a shaker at 180 rpm. Harvesting was performed 3 days after inoculation by centrifugation at 3000 ***g*** for 15 min at 4 °C.

Second, expression was performed in 1.3 L benchtop‐scale bioreactors filled with 1.0 L of fermentation medium. As a starter culture, recombinant *S. lividans* TK23 was grown in shake flask in fermentation medium for 3 days with 10 μg·mL^−1^ thiostrepton at 30 °C and agitated at 180 rpm. After 3 days, the preculture was inoculated overnight from the starter culture at an OD_600_ of 0.02. Cultivation in the fermenter was performed in a DASGIP Parallel Bioreactor (Eppendorf, Hamburg, Germany). The fermentation medium was inoculated with an OD_600_ 0.1 with the preculture. The cultivation temperature was 30 °C, and dissolved oxygen was set to 30%, regulated by the stirrer speed. Before 30% dissolved oxygen was reached, the stirrer was set to 350 rpm. The pH was monitored online and maintained at pH 7.0 with 2 m NaOH and 20% (v/v) H_2_SO_4_. The culture was fed with a 1.5 g·mL^−1^ glucose solution. The feed was initiated after 15 h of incubation at a rate of 1 mL·h^−1^ for 7 h. Subsequently, after 22 h of cultivation, the flow rate was increased to 1.5 mL·h^−1^ until the end of the fermentation. Polypropylene glycol (PPG 2000) was added as an antifoaming agent, regulated by the foam sensor. Fermentation lasted for 2 days for expression of SgAA and 5 days for expression of SgELA. The cultures were harvested by centrifugation for 30 min at 4 °C and 3000 ***g***.

### Protein purification, concentration measurement, and electrophoresis

The harvested cells were resuspended in 100 mm Tris–HCl 150 mm NaCl pH 7.0 to a final volume of 400 mL. The cell lysis was performed by sonication on ice with a 45‐s pulse followed by a 60‐s pause, repeated four times. The cell debris was separated by centrifugation at 16 000 ***g*** for 30 min at 4 °C. The recombinant aminoacylases were purified by Strep‐tag II affinity purification. The column was a 5 mL Strep‐Tactin® SuperFlow® high capacity cartridge. The buffer used for column wash was 100 mm Tris–HCl 150 mm NaCl pH 7.0. For elution of the recombinant protein, 2.5 mm D‐desthiobiotin was added to the wash buffer. The relevant elution fractions were pooled and rebuffered to the wash buffer without desthiobiotin in Vivaspin™ 6 concentrators (10 000 MWCO; Sartorius, Göttingen, Germany). The protein concentrations were measured with the Bradford method [46] using the Roti®‐Nanoquant reagent (Carl Roth, Germany). Gradient gels from 8% to 20% acrylamide content were poured for SDS‐polyacrylamide gel electrophoresis (SDS/PAGE) [47]. The protein molecular weight marker was FastGene® BluEasy Protein Marker (Nippon Genetics Europe, Düren, Germany). Blue native PAGE was performed with SERVAGel™ N4‐16% gels (SERVA, Germany), with reagents from SERVA according to the manufacturer's instructions. The protein marker was SERVA Native Marker, Liquid Mix for BN/CN, and further reference proteins were from Gel Filtration Calibration Kit (High Molecular Weight) from Cytiva (Marlborough, MA, USA). The gels were stained with Coomassie from Rotiphorese® solution (Carl Roth).

### Aminoacylase activity assay

The aminoacylase activity was measured by quantification of the released amino acids with the ninhydrin reaction as described previously [48]. The hydrolytic reactions consisted of 190 μL substrate solution and 10 μL enzyme solution. The reaction course was followed by withdrawing 10 μL samples from the reaction and mixing with 100 μL of EZ Nin:DMSO reagent, heating for 10 min at 99 °C, and diluting 25 μL of the colored sample with 225 μL of 100 mm Na‐borate pH 10.0 for measurement. Standard hydrolytic activity assay for SgAA was performed with 15 mm N‐acetyl‐l‐alanine or N‐acetyl‐l‐methionine in 100 mm Tris–HCl pH 7.0 at 30 °C. The pH of all substrate solutions was measured and adjusted at the respective temperatures. The ninhydrin reaction products of all amino acids were measured at 570 nm, except for the reactions with l‐cysteine and l‐proline, which were measured at 410 nm. The hydrolysis of N_α_‐acetyl‐l‐lysine and N_ε_‐acetyl‐l‐lysine was quantified by reaction with an acidic variant of the ninhydrin reagent mixed with glacial acetic acid (EZ Nin:GAA) and was measured at 460 nm [48]. The hydrolysis of dipeptides was measured in the same way, except a substrate concentration of 7.5 mm was used. One unit of SgAA was defined as the amount of enzyme that hydrolyzes one μmol of N‐acetyl‐l‐alanine per minute under the given conditions, and one unit of SgELA hydrolyzes one μmol of N_ε_‐acetyl‐l‐lysine per minute.

### Biochemical characterization of the aminoacylases

The dependency of SgAA activity on zinc or cobalt ions was investigated by incubating the purified enzyme with 1 mm CoCl_2_ or ZnCl_2_ in 100 mm Tris–HCl at pH 7.0 and 30 °C for 1 h. Likewise, the enzyme was also incubated with the chelating agent ethylenediaminetetraacetic acid (EDTA) at 1 mm concentration. After the incubation period, the hydrolytic activity was determined with 15 mm N‐acetyl‐l‐methionine in 100 mm Tris–HCl pH 7.0 at 30 °C.

The hydrolytic substrate specificity of SgAA was determined with various 15 mm substrates in 100 mm Tris–HCl pH 7.0 with a reaction time of 5 min. The reaction temperature was increased to 50 °C, so that all substrates were solubilized. The substrate specificity of SgELA was determined with the same acetyl‐l‐amino acids as for SgAA at 15 mm in 100 mm Tris–HCl pH 7.0 at 30 °C with a reaction time of 1 h.

The temperature optimum of SgAA was determined by hydrolysis of 15 mm N‐acetyl‐l‐methionine at various temperatures from 20 °C to 70 °C at pH 7.0 in 100 mm Tris–HCl buffer. The temperature stability was assessed by incubation of the enzyme solution at temperatures from 4 °C to 80 °C in the same buffer at the respective temperatures. After 1‐h incubation, residual activity was measured with 15 mm N‐acetyl‐l‐methionine.

The pH dependency of hydrolytic activity of SgAA was determined using the following buffers at 100 mm concentration: Na‐acetate for pH 4.0–5.0, Na‐MES for pH 6.0–7.0, Tris–HCl for pH 6.0–9.0, and Na‐borate for pH 9.0–11.0. The substrate solutions consisted of 15 mm N‐acetyl‐l‐methionine dissolved in these buffers and adjusted at 30 °C. The optimal pH for hydrolysis with SgELA was investigated by preparing 15 mm N_ε_‐acetyl‐l‐lysine in 100 mm Tris–HCl adjusted to pH 6.0–10.0 at 30 °C.

### Biocatalytic synthesis of lauroyl amino acids

For the synthesis of N‐lauroyl‐l‐amino acids, 100 mm lauric acid and 100 mm of all proteinogenic l‐amino acids in 100 mm Tris–HCl buffer at pH 7.0 were used as substrate. For the reaction, 10 μg of enzyme per 0.5 mL reaction was used and the reaction was carried out at 40 °C. The reaction mixtures were analyzed with an HPLC system (S5200 and S2100; Sykam, Fürstenfeldbruck, Germany) equipped with a C18 column (ISAspher 100‐5 C18 BDS column, 5 μm, 4.0 × 250 mm; Isera, Düren, Germany), at a column temperature of 40 °C with the flow rate set to 1 mL·min^−1^. The liquid phase consisted of 80% acetonitrile: 20% H_2_O, 0.1% trifluoroacetic acid, and an isocratic elution was performed. The analytes were detected with a UV Detector (2500 Sykam, Germany) at 210 nm and an ELSD detector (evaporative light scattering detector; ZAM 4000, Schambeck SFD, Bad Honnef, Germany). HPLC‐MS analysis was conducted with a Shimadzu Nexera XR system equipped with a Hitachi LaChrom II column (C18, 5 μm, 4.6 × 250 mm) and a Shimadzu LCMS‐2020‐mass spectrometer. The column temperature was set to 40 °C. A gradient elution was applied starting at 20% acetonitrile and 80% water and 0.1% trifluoroacetic acid and going to 100% acetonitrile in 10 min, which was held for 6 min.

## Results and Discussion

### Cloning and sequence analysis of SgAA and SgELA

**Fig. 1:**
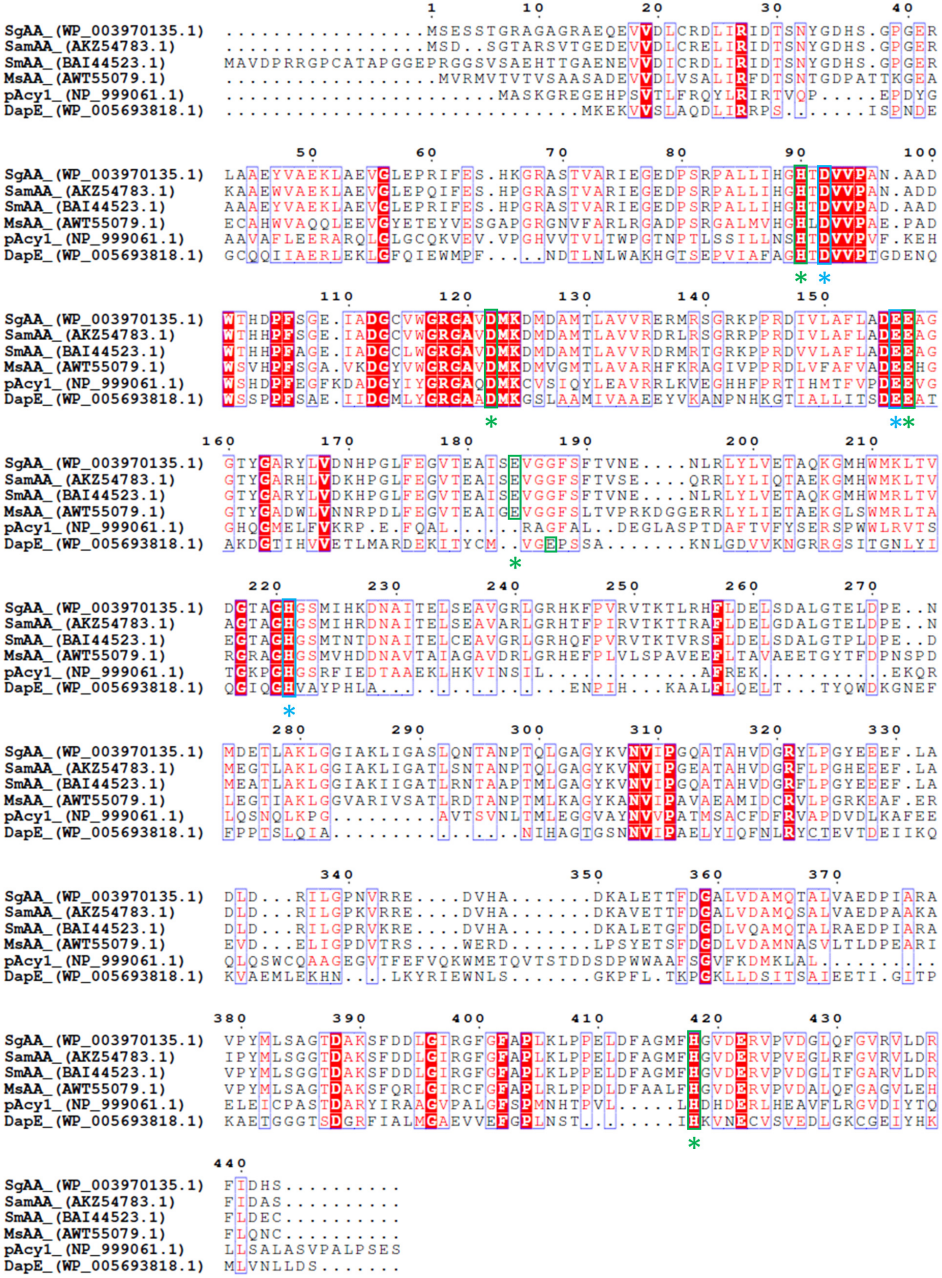
Multiple sequence alignment of SgAA and homologous proteins. SgAA from *S. griseus* (WP 003970135.1), SamAA from *S. ambofaciens* (AKZ54783.1), SmAA from *S. mobaraensis* (BAI44523.1), MsAA from *M. smegmatis* (AWT55079.1), pAcy1 from *sus scrofa* (NP 999061.1), and DapE from *H. influenzae* (WP 005693818.1) are included in the alignment. The alignment was generated with the Clustal W algorithm and displayed with ESPript 3.0. The conserved metal‐binding and catalytic residues are shown with green and blue boxes and asterisks, respectively.

Homologs of the aminoacylases SmAA, SamAA, SmELA, and SamELA were found in the genome of *S. griseus* DSM 40236^T^. The protein sequences were used as baits and the putative aminoacylases SgAA (Accession No. WP_003970135.1) and SgELA (Accession No. WP_069631407.1) were identified with NCBI BLASTp search. The genes were amplified by PCR using genomic DNA from *S. griseus* DSM 40236^T^ as a template and were subsequently cloned into the *S. lividans* expression vector pGH01 and fused with an N‐terminal Strep‐tag II. Sequence identities of SgAA to SamAA (Accession No. AKZ54783.1), SmAA (Accession No. BAI44523.1), MsAA (Accession No. AWT55079.1), pAcy1 (Accession No. NP 999061.1), and HiDapE (Accession No. WP 005693818.1) are 88.3%, 83.7%, 56.5%, 26.5%, and 23.7%, respectively, as determined by the Needleman–Wunsch algorithm [39]. Protein sequence alignment of SgAA with its homologs allowed to identify conserved residues in SgAA, with metal‐binding (H90, D122, E157, E184, and H418) and catalytic (D92, E156, and H221) function (Fig. 1). By using the MEROPS BLAST tool, SgAA can be assigned to the M20A peptidase family in accordance with the homologous enzymes and the identification of conserved residues.

**Fig. 2:**
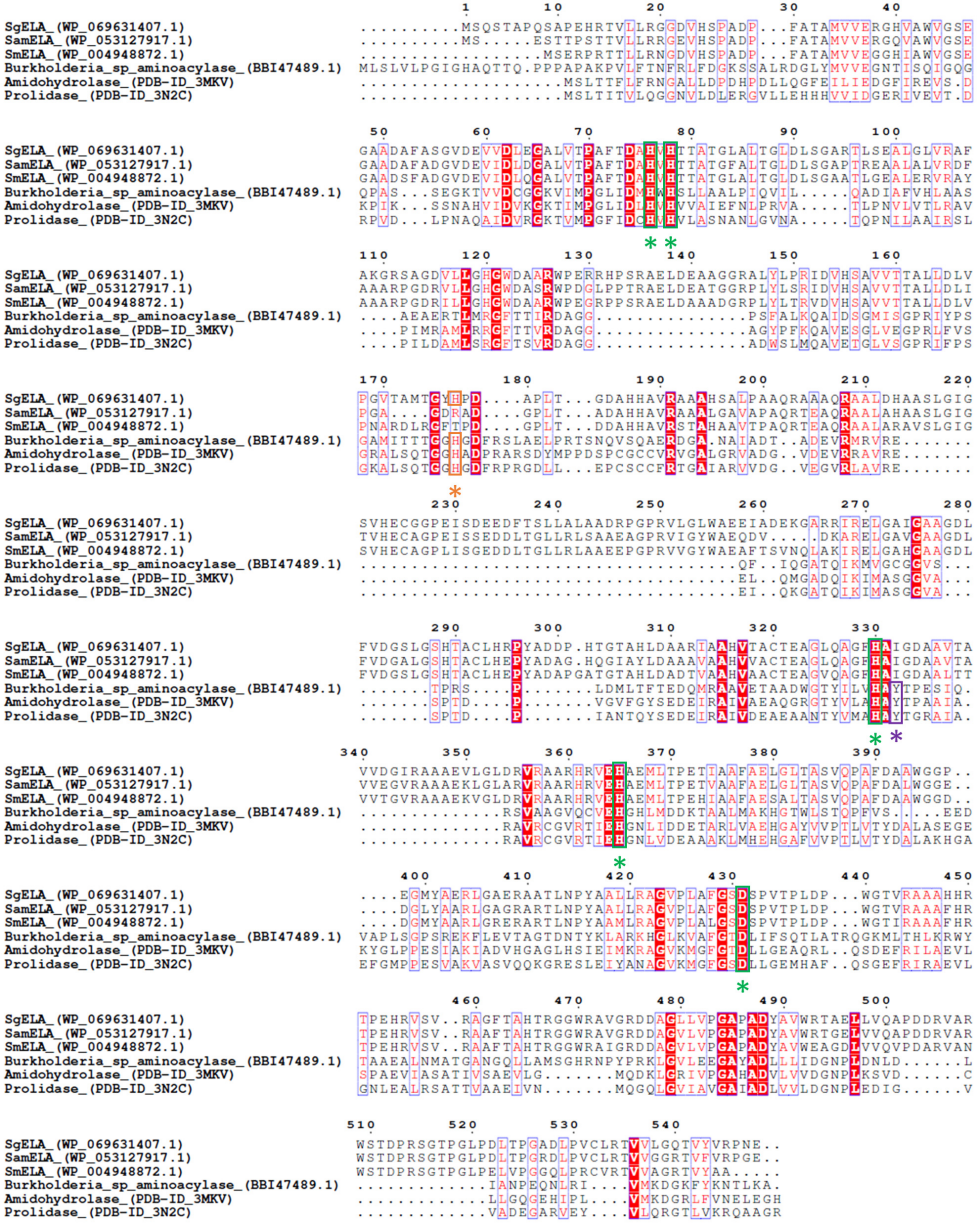
Multiple sequence alignment of SgELA and homologous proteins. SgELA from *S. griseus* (WP_069631407.1), SamELA from *S. ambofaciens* (WP_053127917.1), SmELA from *S. mobaraensis* (WP_004948872.1), aminoacylase from *Burkholderia* species (BBI47489.1), amidohydrolase Sgx9260b (PDB‐ID_3MKV), and prolidase Sgx9260c (PDB‐ID_3N2C) are included in the alignment. The alignment was generated with the Clustal W algorithm and displayed with ESPript 3.0. The conserved metal‐binding residues are shown with green boxes and asterisks. The partly conserved oxyanion hole‐forming histidine described for the amidohydrolase superfamily is highlighted in orange. The partly conserved tyrosine residue that binds the α‐carboxylic group of amino acid substrates in amidohydrolases is highlighted in purple [49].

The sequence of SgELA shows identity to ScELA (GenBank HV688803.1), SamELA (Accession No. WP 053127917.1), and SmELA (Accession No. WP 053127917.1), *Burkholderia* sp. aminoacylase (Accession No. BBI47489.1), amidohydrolase Sgx9260b (PDB 3MKV), and prolidase Sgx9260c (PDB 3N2C) [49] with 79.8%, 79.6%, 75.6%, 21.0%, 21.2%, and 17.8%, respectively. The sequences of these proteins were subjected to MEROPS BLAST, which suggested their classification as M38 metallopeptidases. A protein sequence alignment revealed conserved residues of SgELA for metal‐binding (H76, H78, H330, H365, and D431). The residue H177 of SgELA is conserved among SgELA, *Burkholderia* sp. aminoacylase (Accession No. BBI47489.1), amidohydrolase Sgx9260b (3MKV), and prolidase Sgx9260c (3N2C) and has been described as oxyanion hole‐forming [49]. The tyrosine residue of Sgx9260b and Sgx9260c, which has been assigned the function of binding the α‐carboxylic group of the amino acid substrate, is not conserved in SgELA and the other ε‐lysine aminoacylases (Fig. 2). This might contribute to the missing or low α‐aminoacylase activity of these enzymes. The DNA and protein sequences of the enzymes are shown in the Appendix S1.

### Expression of SgAA and SgELA in shake flasks with *E. coli* and *S. lividans*

The recombinant production of the novel aminoacylases SgAA and SgELA caused difficulties in obtaining soluble enzymes; the enzymes were first expressed in recombinant *E. coli* BL21(DE3) after codon‐optimization according to the bias of *E. coli*. Both enzymes were very prone to the formation of inclusion bodies, regardless of whether a Strep‐tag was attached or not. Overexpression in *E. coli* resulted in the formation of a significant amount of protein as detected by SDS/PAGE, but the recombinant enzymes were present in the insoluble fraction, and no soluble aminoacylase could be obtained by affinity purification (data not shown). This was the case for IPTG induction or lactose autoinduction at all tested temperatures (20–37 °C). The co‐expression of GroEL/S did not lead to soluble protein either. Neither did the use of *E. coli* ArcticExpress (DE3), which constitutively expresses the cold‐adapted chaperonins Cpn60/10 from *Oleispira antarctica*. In a study on the expression of the aggregation‐prone MsAA from *M. smegmatis*, a homolog of SgAA, the abovementioned approaches led to improvement of soluble expression [8]. This leads to the conclusion that the aminoacylases from *S. griseus* DSM 40236^T^ are less suitable for production in *E. coli*. Hence, *S. lividans* TK23 was used as an alternative production host that is genetically closer related to the natural producer. However, the expressed aminoacylases were barely visible as bands after SDS/PAGE and no differences between the Strep‐tag variants were observed. Still small amounts of SgAA NTag and SgELA NTag could be purified via affinity chromatography (not shown). Since the productivity for the aminoacylases was low, a scale‐up from shake flasks to bioreactors was performed.

### Expression and purification of SgAA and SgELA in bioreactors with *S. lividans* TK23

**Fig. 3:**
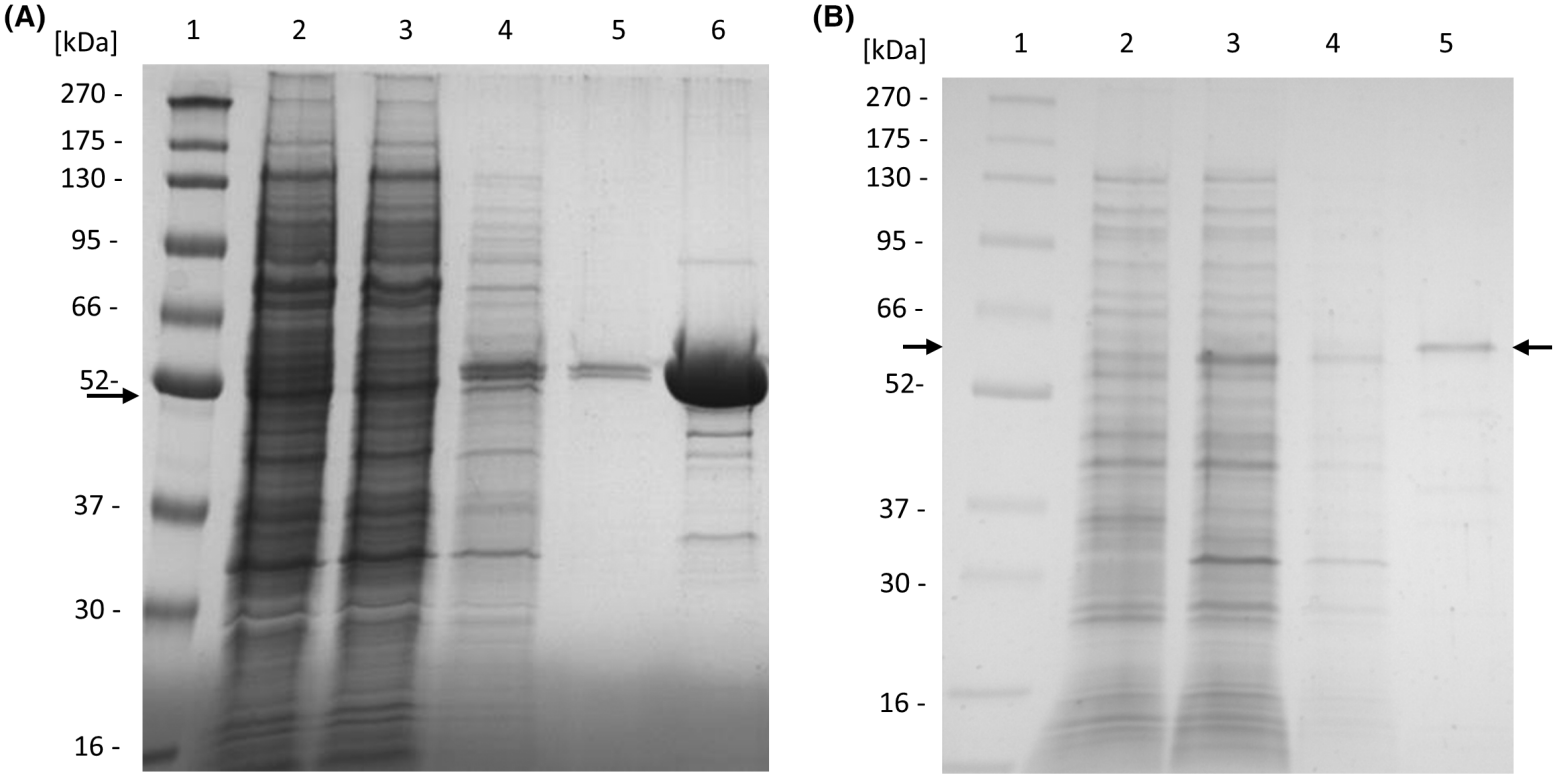
SDS/PAGE analysis of fractions obtained during purification of recombinant *S. griseus* aminoacylases. (A) Affinity chromatography of SgAA. Lane 1: Protein marker (BlueEasy Prestained protein Marker, Nippon Genetics); lane 2: cell‐free extract; lane 3: flow‐through; lane 4 and 5: wash fractions; lane 6: elution of SgAA. (B) Affinity chromatography of SgELA. Lane 1: Protein marker (BlueEasy Prestained protein Marker, Nippon Genetics); lane 2: cell‐free extract; lane 3: flow‐through; lane 4: wash fraction; lane 5: elution of SgELA.

The aminoacylases were eventually produced with recombinant *S. lividans* TK23 transformed with pGH01 SgAA NTag or pGH01 SgELA NTag in bioreactors. After 45 h of growth, SgAA was isolated and purified. The enzyme activity in the cell‐free extract was 11.8 U·mL^−1^ (15 mm N‐acetyl‐l‐alanine, 30 °C, pH 7.0). The cell‐free extract was subjected to Strep‐tag II affinity purification, and the purified enzyme had a specific activity of 65.0 U·mg^−1^ against 15 mm acetyl‐alanine (30 °C, pH 7.0). The SDS/PAGE analysis of the purification of SgAA is shown in Fig. 3, and activities and protein concentrations throughout the purification are summarized in Table S1. In relation to the total protein content, purified recombinant SgAA represents 0.56% of total cellular protein in the cell‐free extract. For SmAA from *S. mobaraensis*, enzyme purified from native producer constituted 0.02% of total protein, while recombinant SmAA made up for 3.24% of total protein in *S. lividans* TK24 [16]. Purified SgAA was subjected to native PAGE (Fig. S1) and was found to be a dimer, as it is described for several homologs of the M20A metallopeptidase family [8, 13, 19]. However, the aminoacylase SmAA from *S. mobaraensis* was described to be a monomeric enzyme as determined by native PAGE [16].

The production of SgELA was performed in bioreactors with an extended cultivation time of 144 h, cells were harvested, and SgELA could be purified from the cell‐free extract in a small amount of 123 μg at a concentration of 16 μg·mL^−1^. The specific activity of purified SgELA was 11.1 U·mg^−1^ against 15 mm Nε‐acetyl‐lysine (30 °C, pH 7.0). The SDS/PAGE analysis of the purification fractions of SgELA is shown in Fig. 3.

### Biochemical characterization of SgAA and SgELA

The purified aminoacylases were biochemically characterized, investigating the effect of metal ions, screening for hydrolytic activity with various acyl amino acids and studying the effect on pH and temperature on activity and stability. The effect of divalent zinc or cobalt ions and the chelating agent EDTA on the enzymatic activity of SgAA was investigated with the purified enzyme. The addition of 1 mm ZnCl_2_ and 1 mm CoCl_2_ reduced the specific activity of SgAA to 50.7% and 90.5%, respectively. Despite the addition of 1 mm of the chelator EDTA, enzyme activity was retained with a specific activity to 107.3%. The homologous enzyme SmAA also showed decreased activity from an excess of Zn^2+^ ions, and EDTA slightly increased the enzyme activity as well [16]. Given the conserved metal‐binding residues of SgAA and the presence of cocatalytic metals in M20A peptidases, it is likely that metal ions are needed for SgAA activity, but at 1 mm ion concentration tested here, an inhibition of aminoacylase activity was observed. The aminoacylase SamAA showed the highest synthetic activity upon the addition of 0.1 mm CoCl_2_, but decreased activity with 0.1 mm ZnSO_4_ or EDTA [5].

The substrate specificity of purified SgAA was determined for the hydrolysis of various acyl amino acids with a concentration of 15 mm at 50 °C and pH 7.0 (Table 1). The enzyme could hydrolyze various acetyl amino acids with a broad specificity regarding the amino acid moiety. Highest specific activities were measured for N‐acetyl‐methionine with 301.1 (± 10.1) U·mg^−1^, N‐acetyl‐alanine with 155.0 (± 4.2) U·mg^−1^, and N_α_‐acetyl‐arginine with 94.4 (± 0.6) U·mg^−1^. In general, SgAA shows higher activities for amino acids with hydrophobic residues, but some amino acids with polar or charged side chains were deacylated as well. Interestingly, in contrast to SmAA and MsAA, which do not hydrolyze N‐acetyl‐proline, SgAA could hydrolyze N‐acetyl‐proline with an activity of 12.0 (± 2.2) U·mg^−1^ under the chosen conditions. Furthermore, SmAA did not hydrolyze acetyl‐glutamic acid, acetyl‐tryptophane, and acetyl‐tyrosine. Comparing to MsAA, the aminoacylase SgAA showed higher activity with N_α_‐acetyl‐arginine, N‐acetyl‐phenylalanine, and N‐acetyl‐tyrosine, which were hydrolyzed with low activity by MsAA under the chosen conditions. Acetyl‐tryptophan was not hydrolyzed by MsAA but was accepted as a substrate by SgAA. The aminoacylase SgAA strongly favors short‐chain acyl residues, showing very low hydrolytic activity with the tested caproyl‐, lauroyl‐, and palmitoyl amino acids. The homologs SmAA and MsAA also favor a short acyl chain length, but activity against various lauroyl amino acids was higher compared with SgAA. No dipeptidase activity of SgAA was detected with alanyl‐phenylalanine or phenylalanyl‐alanine. The purified aminoacylase SgELA showed activity only against 15 mm N_ε_‐acetyl‐lysine with 11.1 U·mg^−1^ (30 °C, pH 7.0). No activity was detected with 15 mm N_α_‐acetyl‐lysine or other 15 mm N_α_‐acyl amino acids which were tested for substrate scope of SgAA (not shown). Hence, the putative classification as an ε‐lysine aminoacylase was confirmed.

**Table 1:** Substrate specificity of SgAA in the hydrolysis of N‐acyl‐l‐amino acids and dipeptides. N‐acyl‐l‐amino acids and dipeptides were used at 15 mm and 7.5 mm concentrations, respectively, in 100 mm Tris–HCl pH 7.0. The reaction temperature was set at 50 °C to solubilize all substrates.

Substrate	Specific activity [U·mg^−1^]
N‐Acetyl‐l‐methionine	301.1 ± 10.1
N‐Acetyl‐l‐alanine	155.0 ± 4.2
N_α_‐Acetyl‐l‐arginine	99.4 ± 0.6
N‐Acetyl‐l‐leucine	43.4 ± 4.2
N‐Acetyl‐l‐valine	25.7 ± 7.0
N‐Acetyl‐l‐phenylalanine	23.5 ± 3.2
N‐Acetyl‐l‐glutamine	16.7 ± 1.0
N‐Acetyl‐l‐asparagine	14.1 ± 0.5
N‐Acetyl‐l‐glycine	13.3 ± 1.7
N‐Acetyl‐l‐proline	12.0 ± 2.2
N‐Acetyl‐l‐threonine	7.7 ± 0.3
N‐Acetyl‐l‐tyrosine	7.5 ± 0.7
N‐Acetyl‐l‐tryptophan	7.0 ± 0.6
N‐Acetyl‐l‐aspartic acid	5.9 ± 0.3
N‐Acetyl‐l‐isoleucine	5.6 ± 0.5
N‐Benzoyl‐l‐alanine	3.3 ± 0.9
N‐Acetyl‐l‐glutamic acid	2.4 ± 0.2
N‐Palmitoyl‐l‐alanine	2.8 ± 0.9
N‐Lauroyl‐l‐methionine	1.4 ± 0.0
N‐Lauroyl‐l‐alanine	1.1 ± 0.0
N‐Caproyl‐l‐glutamine	0.4 ± 0.0
N‐Lauroyl‐l‐glutamine	0.3 ± 0.2
N‐Palmitoyl‐l‐glutamine	0.2 ± 0.2
N‐Lauroyl‐l‐serine	0
N‐Lauroyl‐l‐glycine	0
N‐Lauroyl‐l‐arginine	0
N_α_‐acetyl‐lysine	0
N_ε_‐acetyl‐lysine	0
Phenylalanyl‐alanine	0
Alanyl‐phenylalanine	0

**Fig. 4:**
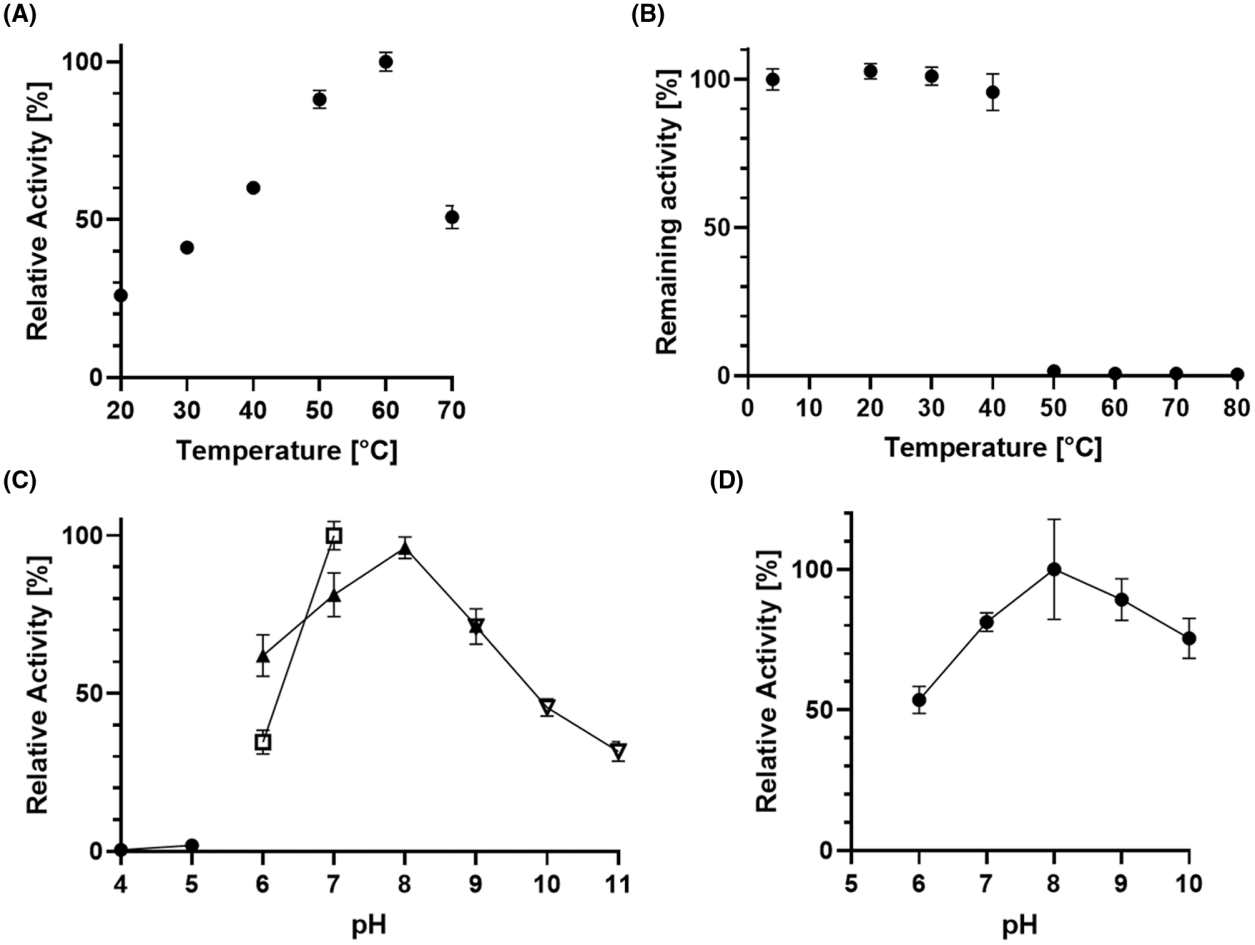
Biochemical characterization of SgAA and SgELA. (A) Temperature optimum of SgAA. Activity was measured at 20–70 °C with 15 mm acetyl‐methionine in 100 mm Tris–HCl pH 7.0. (B) Temperature stability of SgAA. Residual activity after 24 h incubation at temperatures from 4 to 80 °C was measured with 15 mm acetyl‐methionine in 100 mm Tris–HCl pH 7.0 at 30 °C. (C) pH optimum of hydrolysis with SgAA. The activity was measured with 15 mm acetyl‐methionine at 30 °C in various 100 mm buffers. Na‐acetate (●) was used for pH 4.0–5.0, Na‐MES (□) was used for pH 6.0–7.0, Tris–HCl (▲) was used for pH 6.0–9.0, and Na‐borate (▽) was used for pH 9.0–11.0. (D) pH optimum of SgELA in hydrolysis of 15 mm N_ε_‐acetyl‐lysine. All measurements were conducted in triplicate (*n* = 3). The error bars indicate the standard deviations.

The temperature optimum of SgAA was 60 °C with a specific activity of 313 U·mg^−1^ tested with 15 mm acetyl‐methionine in 100 mm Tris–HCl at pH 7.0. The temperature stability was measured after 24‐h incubation at different temperatures and subsequent activity assay with 15 mm acetyl‐methionine in 100 mm Tris–HCl at pH 7.0 and 30 °C. The enzyme was stable up to 40 °C, and at 50 °C and higher temperatures, no remaining hydrolytic activity could be detected. The pH optimum of SgAA was pH 7.0–8.0 at 30 °C (Fig. 4). These characteristics of SgAA are similar to its homologs SmAA and MsAA. The optimal temperature and pH for hydrolysis with SmAA were 50 °C and pH 7.0–8.0, respectively [16]. Highest hydrolytic activity with MsAA was measured at 70 °C and pH 7.0 [8]. Both enzymes showed good stability at 40 °C but decreasing activity when incubated at 50 °C or higher.

The pH optimum of SgELA in hydrolysis of 15 mm N_ε_‐acetyl‐lysine was tested in a range of pH 6.0–10.0 at 30 °C, and the highest activity was measured at pH 8.0. No hydrolytic activity was measured at 50 °C. The enzyme was not stable during storage (in 100 mm Tris–HCl pH 7.0 with 150 mm NaCl) and quickly lost its activity after several hours at 4 °C or −20 °C, so no detailed investigations on stability could be conducted. The homologous enzyme SmELA also had its optimal pH at 8.0 but was significantly more stable, and highest activity was measured at 55 °C [6]. The reasons for the observed instability of SgELA remain unclear, as both SgELA and SmELA were produced with recombinant *S. lividans* cells, and the enzymes are homologs with a sequence identity of 75.6%.

### Biocatalytic synthesis of lauroyl amino acids

**Fig. 5:**
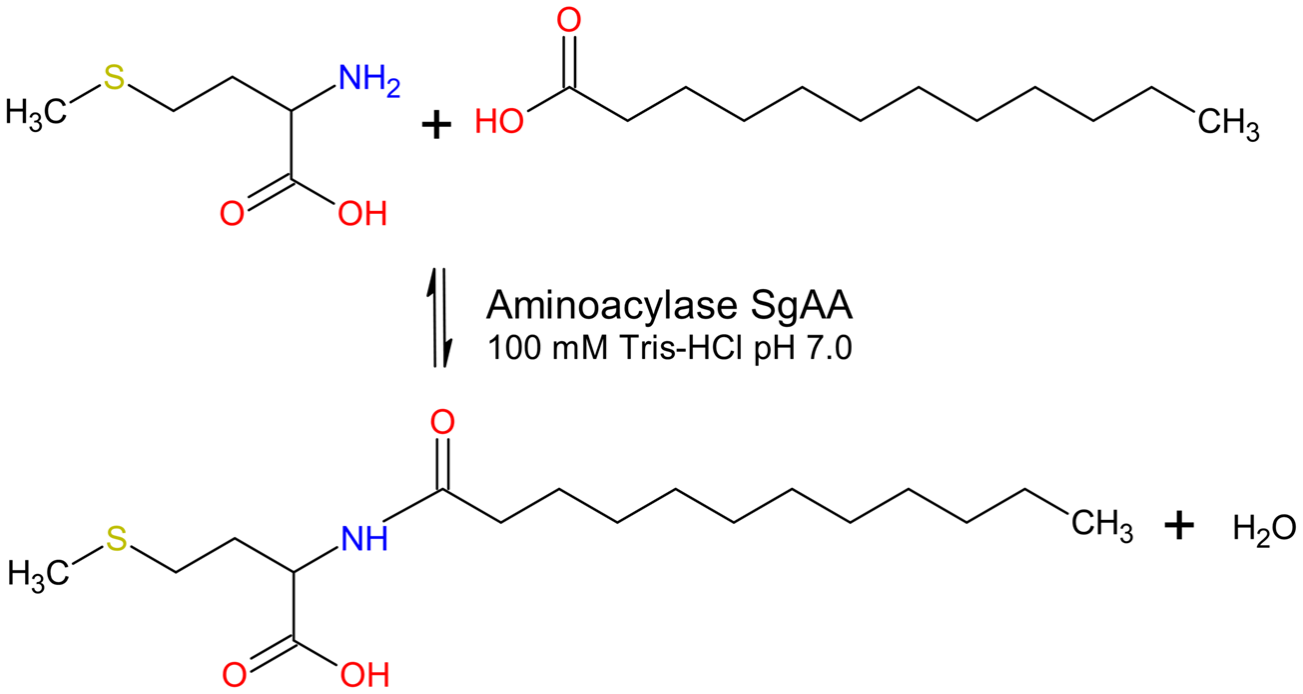
Reaction scheme for production of lauroyl‐methionine catalyzed by SgAA. The condensation reaction was performed with 100 mm l‐methionine and 100 mm lauric acid in 100 mm Tris–HCl at pH 7.0 at 40 °C and 20 μg·mL^−1^ of the aminoacylase SgAA.

Some aminoacylases are capable of amino acid acylation, which makes these enzymes interesting for the synthesis of amino acid‐based surfactants. Hence, we investigated the biocatalytic potential of the aminoacylases from *S. griseus*. In a screen for acylation of all proteinogenic amino acids, the reactions were performed in aqueous buffers and a substrate excess of 100 mm l‐amino acid and 100 mm lauric acid in 100 mm Tris–HCl pH 7.0. After 24 h at 40 °C, and the reaction mixtures were analyzed to detect acylation products. From the reaction conducted with SgELA, no acylation products could be detected under these conditions, whereas the homolog SmELA could produce N_ε_‐lauroyl‐lysine to high yields [25]. SgELA presumably suffered from its low stability. However, the reactions performed with SgAA yielded an acylation product from lauric acid and methionine. A final concentration of 4 mm lauroyl‐methionine was observed (MS spectrum of the product shown in supplements, Fig. S2). The reaction scheme is shown in Fig. 5. The reactions with the remaining proteinogenic amino acids did not result in product formation. Lauroyl‐methionine is a biosurfactant with antioxidative properties and might thus be an interesting additive in cosmetic formulations [50]. The acceptance of further fatty acids is likely and can be investigated in future experiments.

Despite several aminoacylases from *S. mobaraensis* being capable of acylation, no acylation has been reported with SmAA (83.7% sequence identity to SgAA) [16]. In contrast, with SamAA from *S. ambofaciens* (88.3% sequence identity to SgAA), high yields for acylation of various amino acids have been reported. The SamAA enzyme was responsible for the main acylating activity of *S. ambofaciens* [5]. We showed that the newly characterized aminoacylase SgAA is also capable of synthesis of acyl amino acids, specifically lauroyl‐methionine. Interestingly, the homologous MsAA from *M. smegmatis* (56.5% sequence identity) also acylated methionine with highest conversions [8]. The findings are significant for exploring the synthetic potential of streptomycetal M20A aminoacylases, confirming that it is well worth further investigating this sequence space. Future work will focus on the optimization of the synthesis of acyl amino acids with SgAA.

## Conclusions

We present the identification and cloning of aminoacylases SgAA and SgELA from *S. griseus* DSM 40236^T^ that are homologous to aminoacylases from *S. mobaraensis*, *S. ambofaciens*, and *M. smegmatis*. The enzymes were successfully expressed in *S. lividans* TK23 and a protocol for high cell density fermentation in bioreactors was established, which ensures dispersed growth of the bacterial mycelium. Recombinant production and purification of Strep‐tag II‐fused protein in *S. lividans* was performed, which has previously only been reported once for secreted antigens from *M. tuberculosis* [51]. The putative aminoacylase activity could be verified for SgAA and SgELA, and they show characteristics in hydrolysis similar to their homologs. The dimeric enzyme SgAA is a short‐chain acyl aminoacylase with a broad substrate spectrum regarding the amino acid moiety. On the contrary, SgELA is specific of N_ε_‐acetyl‐lysine. Due to its low stability, SgELA did not prove to be suitable for biocatalysis. Future work will deal with the optimization of the conditions for heterologous production. With SgAA, synthesis of lauroyl‐methionine in aqueous buffer was shown, which renders this enzyme interesting for biocatalytic applications and future work to optimize acylation conditions.

## Conflict of interest

The authors declare no conflict of interest.

## Author contributions

GH designed the study. GH conducted cloning and bioinformatic analysis. GH and JP performed the experiments and analyzed the data for protein expression, purification, and biochemical characterization. GH and JP wrote the manuscript. GH, JB, K‐EJ, and PS edited the manuscript. PS and JB supervised the work of GH and JP. PS and JB did fund acquisition. All authors read and approved the final manuscript.

## Supporting information


**Fig. S1.** Native PAGE of SgAA and reference proteins.
**Fig. S2.** Mass spectrum of N‐lauroyl‐l‐methionine produced by SgAA.
**Table S1.** Purification of SgAA from recombinant *S. lividans* culture by Strep‐tag affinity chromatography.

## Data Availability

The datasets supporting the conclusions of this article are included within the article and its additional files.
