# Supplementary material for: Novel aminoacylases from Streptomyces griseus DSM 40236 and their recombinant production in Streptomyces lividans
Source: FEBS Open Bio. 2023 Nov 1;13(12):2224–38. doi: 10.1002/2211-5463.13723 (PMC10699109; doi:10.1002/2211-5463.13723)
Supplement: Supplementary file 1 — Fig. S1. Native PAGE of SgAA and reference proteins. Fig. S2. Mass spectrum of N‐lauroyl‐l‐methionine produced by SgAA. Table S1. Purification of SgAA from recombinant S. lividans culture by Strep‐tag affinity chromatography. [file FEB4-13-2224-s001.docx]

Journal name:

**FEBS Open Bio**

Title:

**Novel aminoacylases from *Streptomyces griseus* DSM 40236 and recombinant production in *Streptomyces lividans***

Author’s names:

Gerrit Haeger^a^, Johanna Probst^a^, Karl-Erich Jaeger^b,c^, Johannes Bongaerts^a^, Petra Siegert^a*^

Addresses:

^a^Institute of Nano- and Biotechnologies, Aachen University of Applied Sciences, 52428 Jülich, Germany

^b^Institute of Molecular Enzyme Technology, Heinrich Heine University Düsseldorf, 52425 Jülich, Germany

^c^Institute of Bio- and Geosciences IBG-1: Biotechnology, Forschungszentrum Jülich GmbH, 52425 Jülich, Germany

Author for correspondence:

Petra Siegert; Heinrich-Mussmann-Str. 1, 52428, Jülich, Germany. E-mail address: siegert@fh-aachen.de (P. Siegert), Tel.: +49 241 6009 53124

**Supplementary material to “Novel aminoacylases from *Streptomyces griseus* DSM 40236 and recombinant production in *Streptomyces lividans*”:**

**DNA and protein sequences for SgAA and SgELA, native PAGE, MS spectrum for lauroyl-methionine**

SgAA NTag DNA sequence from *S. griseus* DSM 40236:

ATGTGGTCCCACCCGCAGTTCGAGAAGTCCGGCAtgagcgagagcagcacgggcagggccggcgccggcagggccgagcaggaggtcgtcgacctctgtcgtgacctgatccggatcgacaccagcaactacggcgaccactcgggccccggcgagcggctcgcggccgagtacgtcgcggagaagctcgcggaggtcggcctggagccgcggatcttcgagtcgcacaagggacgcgcctccaccgtcgcccggatcgagggcgaggacccctcccgcccggcgctgctgatccacggacacaccgacgtcgtcccggccaacgcggcggactggacgcacgacccgttctccggggagatcgcggacggctgcgtgtggggccggggcgcggttgacatgaaggacatggacgcgatgaccctcgcggtcgtccgggagcggatgcgcagcggccgcaagcccccgcgcgacatcgtgctcgccttcctcgcggacgaggaggcgggcggcacgtacggggcccgctatctcgtggacaaccacccgggcctcttcgagggcgtcaccgaggcgatcagcgaggtcggcggcttctccttcaccgtcaacgagaacctgcggctgtatctggtggagaccgcccagaagggcatgcactggatgaagctgaccgtggacggcaccgccggacacggctcgatgatccacaaggacaacgccatcacggagctgtccgaggcggtcgggcggctgggccggcacaagttcccggtgcgggtcaccaagacgctgcggcacttcctggacgagctctccgacgcgctgggcaccgagctggacccggagaacatggacgagacgctcgccaagctcggcggcatcgccaagctcatcggcgcctccctccagaacaccgccaaccccacgcagctcggcgccggctacaaggtcaacgtcatcccgggccaggcgaccgcccacgtggacggccggtacctccccgggtacgaggaggagttcctcgccgacctggaccggatcctcgggcccaacgtccggcgcgaggacgtgcacgcggacaaggccctggagaccacgttcgacggcgcgctggtcgacgccatgcagaccgcgctggtcgccgaggaccccatcgcccgtgccgtgccctacatgctctcggccggcaccgacgccaagtccttcgacgacctgggcatccggggcttcggcttcgccccgctgaagctgccgccggagctggacttcgccggcatgttccacggcgtcgacgagcgcgtcccggtcgacgggctgcagttcggcgtgcgggtgctcgaccggttcatcgaccactcctga

Codon-optimized sequence of SgAA for *E. coli* expression with N- and C-terminal strep-tag shown in bold letters (as ordered from GeneArt, Thermo Fisher):

**ATGTGGTCACATCCGCAGTTTGAAAAAAGCGGT**ATGAGCGAAAGCAGCACCGGTCGTGCCGGTGCAGGTCGTGCAGAACAAGAGGTTGTTGATCTGTGTCGTGATCTGATTCGTATTGATACCAGCAATTATGGTGATCATAGCGGTCCGGGTGAACGTCTGGCAGCAGAATATGTTGCAGAAAAACTGGCAGAAGTTGGTCTGGAACCGCGTATTTTTGAAAGCCATAAAGGTCGTGCCAGCACCGTTGCACGTATTGAAGGTGAAGATCCGAGCCGTCCGGCACTGCTGATTCATGGTCATACCGATGTTGTTCCGGCAAATGCAGCAGATTGGACCCATGATCCGTTTAGCGGTGAAATTGCAGATGGTTGTGTTTGGGGTCGTGGTGCAGTTGATATGAAAGATATGGATGCAATGACCCTGGCAGTTGTTCGTGAACGTATGCGTAGCGGTCGTAAACCGCCTCGTGATATTGTTCTGGCATTTCTGGCAGATGAAGAGGCAGGCGGTACATATGGTGCACGTTATCTGGTTGATAATCATCCGGGTCTGTTTGAAGGTGTTACCGAAGCAATTAGCGAAGTTGGTGGTTTTAGCTTTACCGTGAATGAAAATCTGCGTCTGTATCTGGTGGAAACCGCACAGAAAGGTATGCATTGGATGAAACTGACCGTTGATGGCACCGCAGGTCATggcagcatgattcataaagataacgcgattaccgaactgagcgaagcggtgggccgcctgggccgccataaatttccggtgcgcgtgaccaaaaccctgcgccattttctggatgaactgagcgatgcgctgggcaccgaactggatccggaaaacatggatgaaaccctggcgaaactgggcggcattgcgaaactgattggcgcgagcctgcagaacaccgcgaacccgacccagctgggcgcgggctataaagtgaacgtgattccgggccaggcgaccgcgcatgtggatggccgctatctgccgggctatgaagaagaatttctggcggatctggatcgcattctgggcccgaacgtgcgccgcgaagatgtgcatgcggataaagcgctggaaaccacctttgatggcgcgctggtggatgcgatgcagaccgcgctggtggcggaagatccgattgcgcgcgcggtgccgtatatgctgagcgcgggcaccgatgcgaaaagctttgatgatctgggcattcgcggctttggctttgcgccgctgaaactgccgccggaactggattttgcgggcatgtttcatggcgtggatgaacgcgtgccggtggatggcctgcagtttggcgtgcgcgtgctggatcgctttattgatcatagc**TCCGGATGGAGTCATCCTCAATTCGAAAAAtga**

SgAA protein sequence (Accession no. WP_003970135.1) with N-terminal Strep-tag and linker underlined:

MWSHPQFEKSGMSESSTGRAGAGRAEQEVVDLCRDLIRIDTSNYGDHSGPGERLAAEYVAEKLAEVGLEPRIFESHKGRASTVARIEGEDPSRPALLIHGHTDVVPANAADWTHDPFSGEIADGCVWGRGAVDMKDMDAMTLAVVRERMRSGRKPPRDIVLAFLADEEAGGTYGARYLVDNHPGLFEGVTEAISEVGGFSFTVNENLRLYLVETAQKGMHWMKLTVDGTAGHGSMIHKDNAITELSEAVGRLGRHKFPVRVTKTLRHFLDELSDALGTELDPENMDETLAKLGGIAKLIGASLQNTANPTQLGAGYKVNVIPGQATAHVDGRYLPGYEEEFLADLDRILGPNVRREDVHADKALETTFDGALVDAMQTALVAEDPIARAVPYMLSAGTDAKSFDDLGIRGFGFAPLKLPPELDFAGMFHGVDERVPVDGLQFGVRVLDRFIDHS

SgELA NTag DNA sequence from *S. griseus* DSM 40236:

ATGTGGTCCCACCCGCAGTTCGAGAAGTCCGGCATGAGCCAGAGCACCGCCCCCCAGAGCGCCCCCGAACACCGCACCGTGCTGTTGCGCGGTGGAGACGTCCACAGCCCCGCCGACCCGTTCGCCACCGCGATGGTCGTCGAACGCGGGCATGTCGCCTGGGTCGGGTCCGAGGGGGCCGCCGACGCCTTCGCGAGCGGCGTGGACGAGGTGGTCGACCTCGAAGGCGCCCTGGTCACCCCGGCGTTCACGGACGCCCATGTGCACACCACCGCCACCGGCCTGGCGCTGACGGGGCTCGACCTCTCCGGCGCCCGCACCCTGTCCGAGGCCCTCGGCCTCGTCCGTGCGTTCGCGAAGGGGCGCTCCGCCGGGGACGTTCTGCTCGGACACGGCTGGGACGCCGCCCGCTGGCCCGAGCGGCGCCACCCCTCGCGCGCCGAGCTCGACGAGGCGGCCGGCGGCCGGGCCCTGTACCTGCCGCGGATCGACGTGCACTCCGCGGTCGTCACGACGGCCCTGCTCGACCTCGTCCCCGGCGTCACCGCGATGACCGGCTACCACCCCGACGCTCCGCTCACCGGCGACGCCCACCACGCGGTACGGGCCGCCGCCCACAGCGCGCTCCCGGCCGCCCAGCGGGCGGCCGCGCAGCGCGCCGCCCTCGACCACGCCGCCTCCCTCGGCATCGGCAGCGTGCACGAGTGCGGGGGGCCGGAGATCTCCGACGAGGAGGACTTCACCTCGCTGCTCGCGCTCGCCGCCGACCGGCCGGGGCCGCGCGTCCTCGGCCTCTGGGCCGAGGAGATCGCGGACGAGAAGGGCGCCCGGCGCATCCGCGAACTCGGCGCGATCGGCGCGGCCGGCGACCTGTTCGTCGACGGCTCGTTGGGCTCGCACACCGCCTGCCTGCACCGGCCCTACGCGGACGACCCGCACACCGGCACCGCCCACCTGGACGCCGCCCGGATCGCCGCCCACGTCACCGCCTGCACCGAGGCGGGCCTCCAGGCGGGCTTCCACGCCATCGGCGACGCCGCGGTCACCGCCGTGGTGGACGGGATCCGGGCCGCCGCGGAGGTGCTCGGCCTCGACCGCGTCCGGGCCGCCCGGCACCGCGTCGAACACGCCGAGATGCTCACCCCCGAGACGATCGCCGCCTTCGCCGAACTGGGCCTCACCGCCTCCGTCCAGCCCGCCTTCGACGCCGCCTGGGGCGGGCCCGAGGGGATGTACGCCGAGCGCCTCGGCGCGGAGCGGGCCGCCACGCTCAACCCCTACGCGGCGCTGCTGCGGGCCGGCGTGCCCCTGGCCTTCGGCTCCGACAGCCCGGTCACCCCGCTCGACCCCTGGGGCACGGTCCGCGCCGCCGCCCACCACCGCACCCCGGAGCACCGCGTCTCGGTCCGCGCCGGGTTCACCGCGCACACCCGCGGCGGCTGGCGGGCCGTCGGCCGCGACGACGCGGGCCTCCTGGTGCCCGGCGCCCCGGCCGACTACGCCGTCTGGCGCACCGCCGAACTCCTGGTCCAGGCCCCCGACGACCGGGTCGCCCGCTGGTCCACCGACCCCCGGTCCGGCACGCCCGGCCTGCCGGACCTCACCCCCGGGGCCGACCTCCCCGTCTGCCTGCGGACCGTGGTCCTCGGACAAACGGTCTACGTGCGACCGAACGAGTGA

Codon-optimized sequence of SgELA for *E. coli* expression with N- and C-terminal strep-tag shown in bold letters (as ordered from GeneArt, Thermo Fisher):

**ATGTGGTCACATCCGCAGTTTGAAAAAAGCGGT**ATGAGCCAGAGCACCGCACCGCAGAGCGCACCGGAACATCGTACCGTTCTGCTGCGTGGTGGTGATGTTCATAGTCCGGCAGATCCGTTTGCAACCGCAATGGTTGTTGAACGTGGTCATGTTGCATGGGTTGGTAGCGAAGGTGCAGCAGATGCATTTGCAAGCGGTGTTGATGAAGTTGTTGATCTGGAAGGTGCACTGGTTACACCGGCATTTACCGATGCACATGTTCATACCACCGCAACCGGTCTGGCACTGACAGGTCTGGATCTGAGCGGTGCACGTACCCTGAGCGAAGCACTGGGTTTAGTTCGTGCATTTGCCAAAGGTCGTAGTGCCGGTGATGTGCTGTTAGGTCATGGTTGGGATGCAGCACGTTGGCCTGAACGTCGTCATCCGAGCCGTGCAGAACTGGATGAAGCAGCCGGTGGTCGTGCACTGTATCTGCCTCGTATTGATGTGCATAGCGCAGTTGTGACCACCGCACTGCTGGATCTGGTTCCGGGTGTTACCGCAATGACCGGTTATCATCCTGATGCACCGCTGACCGGTGATGCCCATCATGCAGTTCGTGCCGCAGCACATAGCGCACTGCCTGCAGCACAGCGTGCAGCTGCTCAGCGTGCCGCACTGGATCATGCAGCAAGCTTAGGTATTGGTAGCGTTCATGAATGTGGTGGTCCGGAAATTAGTGATGAAGAAGATTTTACCAGCCTGCTGGCACTGGCAGCAGATCGTCCGGGTCCGCGTGTTTTAGGTCTGTGGGCTGAAGAAATTGCAGATGAAAAAGGTGCACGTCGTATTCGTGAACTGGGTGCAATTGGTGCAGCGGGTGACCTGTTTGTTGATGGTAGCCTGGGTAGCCATACCGCATGTCTGCATCGTCCGTATGCAGATGATCCGCATACCGGCACCGCACATCTGGATGCAGCCCGTATTGCAGCACATGTTACCGCCTGTACCGAAGCAGGTCTGCAAGCAGGTTTTCATGCCATTGGTGATGCCGCAGTTACCGCAGTGGTGGATGGTATTCGTGCAGCAGCCGAAGTTCTGGGCTTAGATCGTGTTCGCGCAGCACGTCATCGTGTTGAACATGCAGAAATGCTGACACCGGAAACCATTGCAGCATTTGCCGAACTGGGTCTGACCGCAAGCGTTCAGCCTGCATTTGATGCAGCATGGGGTGGTCCTGAAGGTATGTATGCAGAACGTCTGGGTGCCGAACGTGCAGCAACCCTGAATCCGTATGCGGCACTGTTACGTGCCGGTGTTCCGCTGGCATTTGGTAGTGATAGTCCGGTGACACCGCTGGATCCGTGGGGCACCGTACGTGCAGCGGCACATCATCGTACCCCTGAACATCGTGTTAGCGTTCGTGCAGGTTTTACCGCACATACCCGTGGTGGTTGGCGTGCAGTTGGTCGTGATGATGCCGGTCTGCTGGTTCCTGGTGCACCGGCAGATTATGCAGTTTGGCGTACCGCAGAACTGCTGGTGCAGGCACCGGATGATCGTGTTGCACGTTGGAGCACCGATCCGCGTAGCGGTACACCGGGTCTGCCGGATCTGACCCCTGGTGCCGATCTGCCTGTTTGTCTGCGTACCGTTGTTCTGGGTCAGACCGTTTATGTTCGTCCGAATGAA**AGCGGTTGGAGCCATCCTCAGTTCGAGAAAtga**

SgELA protein sequence (Accession no. WP_069631407.1) with N-terminal Strep-tag and linker underlinded:

MWSHPQFEKSGMSQSTAPQSAPEHRTVLLRGGDVHSPADPFATAMVVERGHVAWVGSEGAADAFASGVDEVVDLEGALVTPAFTDAHVHTTATGLALTGLDLSGARTLSEALGLVRAFAKGRSAGDVLLGHGWDAARWPERRHPSRAELDEAAGGRALYLPRIDVHSAVVTTALLDLVPGVTAMTGYHPDAPLTGDAHHAVRAAAHSALPAAQRAAAQRAALDHAASLGIGSVHECGGPEISDEEDFTSLLALAADRPGPRVLGLWAEEIADEKGARRIRELGAIGAAGDLFVDGSLGSHTACLHRPYADDPHTGTAHLDAARIAAHVTACTEAGLQAGFHAIGDAAVTAVVDGIRAAAEVLGLDRVRAARHRVEHAEMLTPETIAAFAELGLTASVQPAFDAAWGGPEGMYAERLGAERAATLNPYAALLRAGVPLAFGSDSPVTPLDPWGTVRAAAHHRTPEHRVSVRAGFTAHTRGGWRAVGRDDAGLLVPGAPADYAVWRTAELLVQAPDDRVARWSTDPRSGTPGLPDLTPGADLPVCLRTVVLGQTVYVRPNE

Table S1: Purification of SgAA from recombinant S. lividans culture by Strep-tag affinity chromatography. Aminoacylase activity was measured with 15 mM alanine in 100 mM Tris-HCl pH 7.0 at 30 °C. One U of SgAA hydrolyzes one µmol of N-acetyl-L-alanine per minute.

| **Sample** | **Sample volume [ml]** | **Protein concentration [mg/ml]** | **Total protein [mg]** | **Enzyme activity [U/ml]** | **Total activity**  **[U]** | **Specific activity [U/mg]** |
| --- | --- | --- | --- | --- | --- | --- |
| Cell-free extract | 400 | 6.1 | 2440 | 11.8 | 4720 | 1.94 |
| Flow-through | 400 | 5.2 | 2360 | 8.3 | 3320 | 1.59 |
| Wash fraction 1 | 50 | 0.19 | 9.75 | 5.8 | 290 | 29.58 |
| Wash fraction 2 | 50 | 0.046 | 2.3 | 1.3 | 65 | 29.25 |
| Wash fraction 3 | 50 | 0.023 | 1.15 | 0.8 | 40 | 35.28 |
| Elution | 25 | 0.55 | 13.75 | 35.8 | 895 | 65.04 |


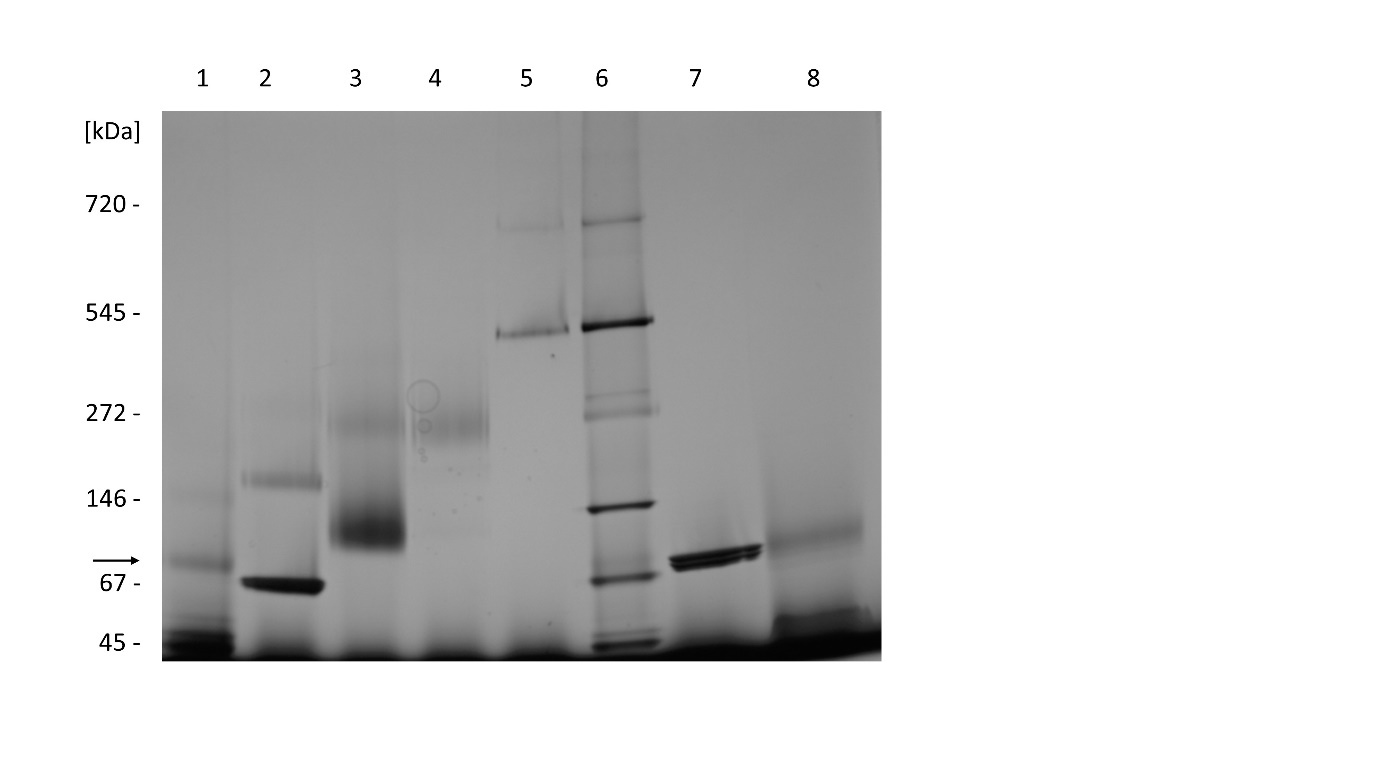


Figure S1: Native PAGE of SgAA and reference proteins.

lane 1: ovalbumin (44 kDa); lane 2: BSA (67 kDa); lane 3: conalbumin (75 kDa); lane 4: aldolase (158 kDa); lane 5: ferritin (440 kDa); lane 6: protein marker (SERVA Native Marker, Liquid Mix for BN/CN); lane 7: SgAA; lane 8: CsAga (α-glutamine aminoacylase from Corynebacterium striatum Ax20; 96 kDa dimeric/48 kDa monomeric; heterologously expressed; unpublished data)


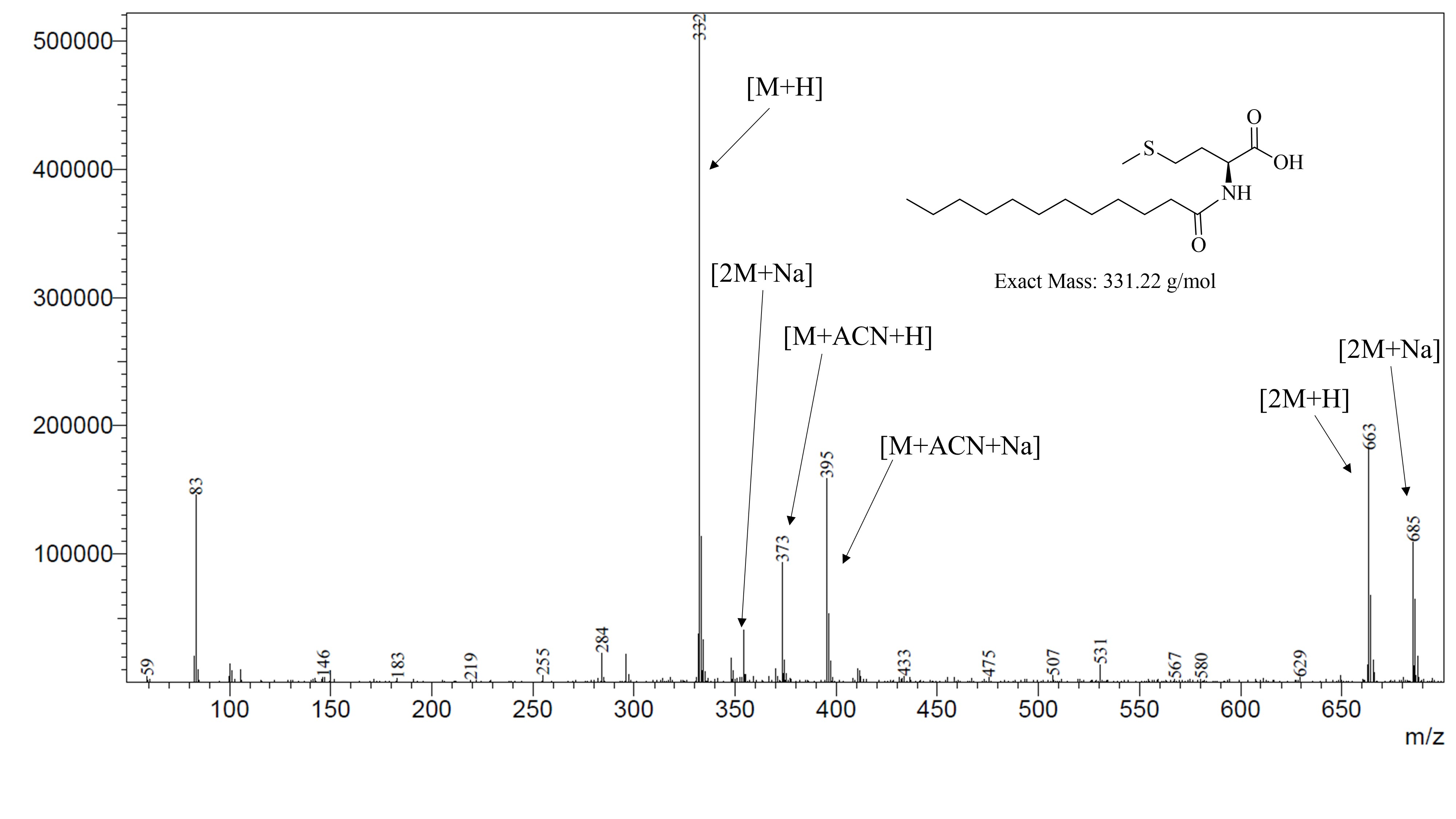


Figure S2: Mass-spectrum of N-lauroyl-L-methionine produced by SgAA.
